# Early release of high-mobility group box 1 (HMGB1) from neurons in experimental subarachnoid hemorrhage *in vivo* and *in vitro*

**DOI:** 10.1186/1742-2094-11-106

**Published:** 2014-06-12

**Authors:** Qing Sun, Wei Wu, Yang-Chun Hu, Hua Li, Dingding Zhang, Song Li, Wei Li, Wei-De Li, Biao Ma, Jian-Hong Zhu, Meng-Liang Zhou, Chun-Hua Hang

**Affiliations:** 1 Department of Neurosurgery, Jinling Hospital, School of Medicine, Nanjing University, Nanjing, Jiangsu Province, China; 2 Department of Neurosurgery, School of Medicine, Southern Medical University(Guangzhou), Jinling Hospital, Nanjing, Jiangsu Province, People's Republic of China; 3 Department of Neurosurgery, Jinling Hospital, 305 East Zhongshan Road, 210002 Nanjing, People's Republic of China

**Keywords:** HMGB1, SAH, Early brain injury

## Abstract

**Background:**

Translocation of high-mobility group box 1 (HMGB1) from nucleus could trigger inflammation. Extracellular HMGB1 up-regulates inflammatory response in sepsis as a late mediator. However, little was known about its role in subarachnoid hemorrhage-inducible inflammation, especially in the early stage. This study aims to identify whether HMGB1 translocation occurred early after SAH and also to clarify the potential role of HMGB1 in brain injury following SAH.

**Methods:**

Sprague-Dawley (SD) rats were randomly divided into sham group and SAH groups at 2 h, 12 h and on day 1, day 2. SAH groups suffered experimental subarachnoid hemorrhage by injection of 0.3 ml autoblood into the pre-chiasmatic cistern. Rats injected by recombinant HMGB1(rHMGB1) solution were divided into four groups according to different time points. Cultured neurons were assigned into control group and four hemoglobin (Hb) incubated groups. Mixed glial cells were cultured and stimulated in medium from neurons incubated by Hb. HMGB1 expression is measured by western blot analysis, real-time polymerase chain reaction (PCR), immunohistochemistry and immunofluorescence. Downstream nuclear factor kappa B (NF-κB) subunit P65 and inflammatory factor Interleukin 1β (IL-1β) were measured by western blot and real-time PCR, respectively. Brain injury was evaluated by cleaved caspase-3 staining.

**Results:**

Our results demonstrated HMGB1 translocation occurred as early as 2 h after experimental SAH with mRNA and protein level increased. Immunohistochemistry and immunofluorescence results indicated cytosolic HMGB1 was mainly located in neurons while translocated HMGB1 could also be found in some microglia. After subarachnoid injection of rHMGB1, NF-κB, downstream inflammatory response and cleaved caspase-3 were up-regulated in the cortex compared to the saline control group. *In-vitro*, after Hb incubation, HMGB1 was also rapidly released from neurons to medium. Incubation with medium from neurons up-regulated IL-1β in mixed glial cells. This effect could be inhibited by HMGB1 specific inhibitor glycyrrhizic acid (GA) treatment.

**Conclusion:**

HMGB1 was released from neurons early after SAH onset and might trigger inflammation as an upstream inflammatory mediator. Extracellular HMGB1 contributed to the brain injury after SAH. These results might have important implications during the administration of specific HMGB1 antagonists early in order to prevent or reduce inflammatory response following SAH.

## Introduction

Subarachnoid hemorrhage (SAH), especially aneurysm subarachnoid hemorrhage, is a life-threatening disease of the central nervous system (CNS). The incidence of SAH is about 22.5 cases per 100,000 in the population according to a World Health Organization study [1]. Although it has relatively low incidence, the early age of onset and poor outcome results in a many life-years lost. Toll like receptors (TLRs), myeloid differentiation primary response protein 88 (MyD88), NF-κB, IL-1β and TNF-α have been proved to participate in the damaging inflammation process after SAH [2-5]. Moreover, clinical studies have shown that increased levels of pro-inflammatory factors in cerebrospinal fluid (CSF) and serum of SAH patients are associated with poor outcome [6,7]. Elevated inflammatory factors contribute to the breakdown of the blood brain barrier (BBB), brain edema, neuroglia cells apoptosis and death [8]. Administration of the antagonists of the pro-inflammatory factors confers a neuroprotective effect in library experimental studies [2,5]. However, how the inflammatory response is initiated and upstream of the inflammation response is still poorly investigated.

High-mobility group box 1(HMGB1), as a nonhistone protein binding with DNA, is widely expressed in the nucleus of nearly all eukaryotic cells, including brain cells, and stabilizes nucleosome formation, facilitates gene transcription [9]. Growing evidence shows that HMGB1 is passively released from necrotic cells or actively secreted from immune cells or non-immune parenchymal cells under various pathological conditions [9]. Extracellular HMGB1 serves as alarmin or damage-associate molecular pattern (DAMP) that mediates cross-talk between damaged cells and relative healthy cells and triggers inflammatory response after interaction with TLR2, TLR4 and receptors for advanced glycation end-products (RAGE) [10,11]. Extracellular HMGB1 has been regarded as a late inflammatory mediator in sepsis and as an early mediator in ischemia-inducible models [10,12]. Individual research into HMGB1 in the late stage of SAH has shown that HMGB1 is highly expressed in a day-5 group in brain stem tissue in the rabbit SAH model [13]. However, previous study suggested that brain parenchymal cells were damaged in the early period after SAH onset [14]. Further, HMGB1 passive translocation usually occurred in the damaged cells [9]. Thus, we supposed that the HMGB1 might translocate early from nucleus to cytoplasm after SAH. Thus, this study aims to identify whether HMGB1 translocation occurred early after SAH and also to detect the expression level of HMGB1 in the early stage and clarify the potential role of HMGB1 in brain injury following SAH.

## Method and material

### Animal preparation

Male Sprague-Dawley rats (280 to 320 g) were purchased from the Animal Center of Jinling Hospital. The rats were raised in a 12-h dark-light cycle with free access to food and water. All procedures were approved by the Animal Care and Use Committee of Nanjing University and conformed to Guide for the Care and Use of Laboratory Animals by National Institutes of Health. Forty-five animals were divided randomly into a sham group and SAH groups at 2 h and 12 h, and on day 1 and day 2 respectively (nine rats per group). Six rats from each group were randomly selected for the analysis of western blot and real-time PCR. In the following step, another 27 rats were prepared for immunohistochemical and immunofluorescence study in the sham group, the 2-h, and the day-1 group (nine rats per group). Six rats each of the selected groups were randomly chosen and sacrificed for immunohistochemical and immunofluorescence study. As for subarachnoid injection of recombinant HMGB1 (rHMGB1), 45 rats were randomly divided into a control group and rHMGB1 injection groups including 2-h, 12-h, day-1 and day-2 groups (9 rats per group). Meanwhile, another 18 rats were prepared for immunofluorescent analysis (9 rats in the control and day-1 group, respectively).

### SAH model

The prechiasmatic injection model was used [15]. Briefly, after intraperitoneal anesthesia with pentobarbital sodium (50 mg/kg) (Sigma, St Louis, MO, USA),then they were positioned prone in a stereotactic frame. After careful disinfection, a midline scalp incision was made and a 1-mm hole was drilled 7.5 mm anterior to the bregma in the midline, at an angle of 30°E caudally. Then they were positioned supine. After careful disinfection again we used an insulin injection needle (BD Science, Franklin Lakes, New Jersey, USA) to obtain 300 μl blood of themselves from femoral artery. The needle was advanced 11 mm into the prechiasmatic cistern through this burr hole, and the 300 μl blood was injected into the prechiasmatic cistern over 20s. Sham rats experienced the same procedure except for injection of 300 μl blood. Cerebral blood flow was monitored for 45 minutes and 60 minutes after SAH. After completing these procedures, 1 ml of 0.9% NaCl was injected subcutaneously to prevent dehydration and the rats were arranged in the recovery cage. It took about 30 minutes to one hour to reach recovery. After the rats started to move around and eat some semi-fluid food, they were returned to their clean and new cages and housed at 23 ± 1°C.

### Subarachnoid prechiasmatic injection of recombinant HMGB1

Prechiasmatic injection animal models were prepared as mentioned above and injected with 150 μl recombinant HMGB1 (rHMGB1) saline solution. RHMGB1 extracted from Human Embryonic Kidney 293 cells (HEK-293) was prepared from Novoprotein(catalog number: C357, purity >95%, PH 7.4, Summit, NJ, USA). The content of endotoxion was tested by the Novoprotein Company and found to be less than 0.1 ng/μg. This result was also confirmed by our endotoxin Limulus amebocyte lysate test (catalog number: KC48, Chinese Horseshoe Crab Reagent Maunfactory, Xiamen, China). Western blot analysis was designed to exclude Histone 3 protein contamination. 50 μg rHMGB1 was diluted to 1,500 μl(33 μg/ml) with saline and sterilized by filtration through a 0.22-μm sterile filter in case of bacterial contamination. The dose of rHMGB1 was determined according to Qiu’s research (3.3 μg rHMGB1/kg) [16] and adjusted the total volume of injection to be 150 μl. Rats in the control group were injected with 150 ul saline. Tissue was prepared for western blot and immunofluorescent analysis.

### Perfusion-fixation and tissue preparation

**Figure 1:**
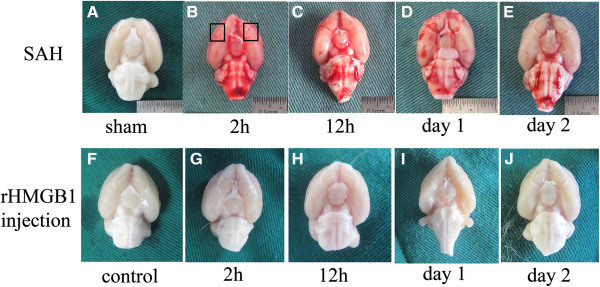
**Schematic observation of the brain after surgery.** Top: rat brains harvested at different times after subarachnoid hemorrhage (SAH) onset. Rat brains in the sham group were harvested one day after the surgical procedure **(A)**. We can conclude that blood clot in the subarachnoid space gradually disappeared with time **(B **to **E)**. Bottom: rat brains harvested at different times after subarachnoid injection of recombinant High-mobility group box 1 (rHMGB1) saline **(G **to **J)**. Rats in the control group were injected with 0.9% saline, and sacrificed one day after the surgical procedure **(F)**.

Animals were sacrificed according to the time points of different groups. In our pilot study, we found that there was no statistical difference in any detected variables among sham groups at any time point (data not shown). Therefore, animals in the sham group were sacrificed at 24 h after the sham operation. Animals were anesthetized as above, and perfused through the left cardiac ventricle with 0.9% a NaCl solution until effluent from the right atrium was clear. Animals that had obvious clots in the prechiasmatic cistern were selected to further analyze. The temporal lobe tissue (black box in Figure 1B), which was near the hematoma, was harvested on ice after blood clots on the tissue were carefully cleared. The tissue was stored at -80°C till further use for western blot, real-time PCR. For immunohistochemistry and immunofluorescence study, the rats were perfused with 0.9% NaCl solution followed by 4% buffered paraformaldehyde. A coronal block cut from 4 mm to 6 mm and 6 mm to 8 mm anterior to the groove between forebrain and cerebellum was prepared and immersed in 4% buffered paraformaldehyde overnight and then embedded in paraffin for immunohistochemistry study and frozen in optimal cutting temperature (OCT) medium for immunofluorescence study, respectively.

### Primary cortical neuron culture, hemoglobin (Hb)-incubated neuron injury model and experimental design

The primary cortical neuron culture was prepared and cultured as per the established technique in our laboratory. Specifically, timed-pregnant female rats (16 to 18 days gestation) were sacrificed with deep anesthesia, and put in 75% alcohol disinfectant for sterilizing. Then 10 to 14 embryos were removed by Caesarean section using sterile techniques. The cortex was separated with the aid of a dissection microscope and rinsed with pre-cooling PBS and treated by 0.1% trypsin for 5 minutes at 37°C, and then the supernatant containing trysin was discarded and washed by pre-cooling PBS. Subsequently, cells were triturated with fire-polished glass pipettes. Then the neuron suspension was filtered through a 22 μm-filter into a 15 ml conical tube and sedimented at 1500 r/minute for 5 minutes at 4°C. After centrifugation, cells were resuspended in neurobasal media with B27 (GIBCO, Rockville, MD, USA) plus antibiotics and were dissociated by repeated pipetting through a 1-ml blue pipette tip. Then the cells were planted at approximately 100 × 10^4^ cells per well in 6-well ploy-D-lysine-coated plates. Cultures were maintained at 37°C in a humidified atmosphere of 5% CO_2_ and 95% air. Subsequently, half of the medium was replaced every 2 days during the first 8 days *in vitro*. The cultures were used on day 8when the cultures were essentially free of astrocytes. Hemoglobin (Hb) provided reactive oxygen species (ROS) and heme, which caused neuron cell injury. Thus, Hb incubation in cultured neurons is often used as a neuron injury model in the researches of hemorrhage disease, including SAH. In this study, this model was also employed as the experimental SAH in the *in vitro* model. Hb (sigma, St. Louis, MO, USA) were prepared and resolved into 10 μM with culture medium and sterilized by filtration through a 0.22-μm sterile filter. Then the neurons were treated with Hb at a concentration of 10 μM, which was determined from prior studies [17]. After 4, 8, 16 and 24 h, the media of neurons were concentrated for protein analysis and cultured neurons were arranged for immunofluorescence staining.

### Primary mixed glial cells culture and cell medium stimulation experimental design

Primary mixed glial cells cultures were prepared as previous study [10]. Briefly, cerebral hemispheres of 1- to 3-day-old postnatal rat brains (Sprague-Dawley rats) were separated with the aid of a dissection microscope and rinsed with pre-cooling PBS and treated by 0.125% trypsin for 5 minutes at 37°C, and then DMEM containing 10% FBS(Hyclone, Logan, Utah, USA) were added to stop the digestion process. Subsequently, cells were triturated by repeated pipetting through a 1-ml blue pipette tip. Then the suspension was filtered through a 22 μm-filter into a 15-ml conical tube and sedimentedat 1,500 r/minute for 5 minutes at 4°C. After centrifugation, cells were resuspended and planted at approximately 100 × 10^4^ cells per well in 6-well plates in DMEM (Hyclone, Logan, Utah, USA) containing 10% FBS(Hyclone, Logan, Utah, USA). Culture media were renewed after 24 h and then twice per week. After 1 week, cells were subjected to different treatments.

Cell medium preparation: neuron cells were cultured as was described above. After incubation with neurobasal medium containing 20 μmol Hb for 2 h, the medium was removed and replaced with fresh DMEM. After neurons with DMEM were cultured for 22 h, the DMEM medium was collected as the neuron medium. The control medium was prepared from neurons treated with neurobasal containing 0 μmol Hb and incubated with DMEM medium for 22 h.

Groups and experiment design: cultured mixed glial cells were arranged into three groups. The control group: mixed glial cells treated with control medium; the medium group: mixed glial cells treated with neuron medium; the glycyrrhizic acid (GA) group: after mixed glial cells were treated with neuron medium, GA (Sigma, catalog number:50531, purity >95%, St. Louis, MO, USA) diluted in PBS and adjusted PH to 7.4, then added to medium, the final concentration of GA in medium was 2 mM), a special inhibitor of HMGB1 was added in the medium to silence the activity of HMGB1 [18,19]. Mixed glial cells in all the groups were cultured for another 24 h. Then, glial cells were collected for real-time PCR analysis.

### Preparation of tissue protein for western blot analysis

#### Total protein extraction

Proper size of tissues (50 ~ 100 mg) were completely homogenized using buffer and centrifuged at 14,000 × g for 15 minutes at 4°C. The supernatant was collected as the total protein extraction of tissue.

#### Cytosolic/nuclear fraction extraction

Rat brain-tissue cytosolic/nuclear fraction extraction was performed following the methods used in our laboratory [20]. The brain tissue (about 100 mg) was homogenized in 1 ml ice-cold buffer A composed of 10 mM HEPES (pH 7.9), 2 mM MgCl_2_, 10 mM KCl,0.1 mM EDTA, 1 mMdithiothreitol (DTT) and 0.5 mM phenyl-methylsulfonyl fluoride (PMSF) (all from Sigma Chemical Co).The homogenate was incubated on ice for 20 minutes, and then 30 μl of 10% NonidetP-40 solution was added (Sigma, St. Louis, MO, USA); the mixture was vortexed for 30 s and spun by centrifugation for 10 minutes at 5,000 g, 4°C. The cytosolic fraction extracts were collected and stored at -80°C for western blot analysis. The crude nuclear pellets were suspended in 200 μl ice-cold buffer B containing 20 mM HEPES (pH 7.9), 25% (v/v) glycerol, 1.5 mM MgCl_2_, 20 mMKCl, 0.1 mM EDTA, 0.5 mM PMSF, and 1 mM DTT, and incubated on ice for 30 minutes with intermittent mixing and centrifuged at 12,000 g at 4°C for 15 minutes. The supernatant containing nuclear proteins was collected and stored at -80°C for western blot analysis. Protein concentration was determined using a bicinchoninic acid assay kit with bovine serum albumin as the standard (Pierce Biochemicals, Rockford, IL, USA).

### Western blot analysis

For western blot analysis, an equal volume of 5 × SDS sample buffer was added to the protein extraction, and the samples were then boiled for 5 minutes. Samples (70 μg per lane) were subjected to electrophoresis 10% SDS-polyacrylamide gels for 30 minutes at 80 V followed by 100 minutes at 110 V and then transferred onto polyvinylidenefluoride (PVDF) for 2 h at 200 mA. The membrane was blocked with 5% defatted milk for 2 h at room temperature, then incubated with primary antibodies at 4°C with gentle shaking overnight. We used anti-HMGB1 monclonal antibody (Catalog no. 2600-1, Epitomics, Inc., Burlingame, CA, USA, diluted 1 : 5000), anti-NF-κB(P65 subunit) (Santa cruz, USA, 1:200 dilution), anti-Histone 3 (Cell signaling technology, Beverly, MA, USA 1:1000 dilution)and β-actin (Catalog no.AP0060, Bioworld, USA, 1: 3000 dilution) After that the membrane was washed for 10 minutes each for four times in TBS + Tween 20 (TBST), followed by incubated in the appropriate HRP-conjugated secondary antibody (Catalog no. BS13278, Bioworld, USA, diluted 1:5000 in TBST) for 2 h at room temperature. The blotted protein bands were visualized by enhanced chemiluminescence (ECL) western blot detection reagents (Catalog number NCI5079, Thermo Scientific, Rockford, IL, USA) and were exposed to x-ray film. Relative changes in protein expression were estimated from the mean pixel density using UN-SCAN-IT, normalized to β-actin, and calculated as target protein expression/β-actin expression ratios.

### RNA isolation and quantitative real-time PCR

Rat brain tissues were isolated using TRIzol Reagent (TAKARA Biotechnology, Japan) as per the manufacturer’s instructions. The concentration of the RNA was determined by spectrophotometric analysis (OD260/280). The quantity of RNA was measured using OD260. The isolated RNA was stored at-80°C until analyzed. RNA was reverse-transcribed to cDNA using Reverse Transcriptase Reagent (TAKARA Biotechnology, Japan) and oligodT primers. Quantitative real-time PCR analysis was performed using the Agilent Technologies Stratagene Mx3000P real-time PCR system (Genetimes Technology, Inc), applying real-time SYBR Green PCR technology. The reaction mixtures contained 1 μl cDNA, 12.5 μl SYBR Green (TAKARA Biotechnology, Japan), 1 μl of each forward and reverse primer (10 μM) and nuclease-free water to a final volume of 25 μl. The primers were synthesized by Life Technologies (Invitrogen, Shanghai, China) and the sequences used were from a database at NCBI for rat HMGB1, TNF-Θ, IL-1β and β-actin. HMGB1 forward and reverse primers were 5’-ATGGGCAAAGGAGATCCTA-3’ and 5’-ATTCATCATCATCATCTTCT-3’; TNF-Θ forward and reverse primers were 5’-TGCCTATGTCTCAGCCTCTTC-3’ and 5’-GAGGCCATTTGGGAACTTCT-3’; IL-1β forward and reverse primers were 5’-TGAGCACCTTCTTTTCCTTCA-3’ and 5’-TTGTCTAATGGGAACGTCACAC-3’; β-actin forward and reverse primers were 5’-AGGGAAATCGTGCGTGAC-3’ and 5’-CGCTCATTGCCGATAGTG-3’. After 95°C for 30 s, 40 PCR cycles were performed; each consisting of a denaturation step (95°C, 5 s) and an annealing step (60°C, 30 s). Total RNA concentrations from each sample were normalized by quantity of β-actin mRNA, and the expression levels of target genes were evaluated by ratio of the number of target mRNA to β-actin mRNA. All samples were analyzed in triplicate.

### Administration of propidium iodide and detection of propidium iodide-positive cells

Propidium iodide (PI), 10 mg/ml (Sigma, St Louis, MO, USA) was diluted in 0.9% NaCl and 1 mg/kg was administered 1 h prior to sacrifice by intraperitoneal injection in a total volume of not more than 100 μl. Brain tissue was fixed with 4% paraformaldehyde overnight and dipped in 20% saccharose PBS for 2 days and then in 30% saccharose PBS for another 2 days to remove water in the tissue. For detection of the relationship between PI-labeled and HMGB1-positive cells, sections 6 μm in thickness were sliced and blocked with 5% normal FBS in PBS containing 0.1% Triton X-100 for 2 h at room temperature prior to incubation with anti-HMGB1 antibody(diluted 1:500, Epitomics, Burlingame, CA, USA) overnight at 4°C. After sections were washed three times with PBS for 45 minutes, they were immunolabeled with secondary antibody (Alexa Fluor 488 diluted 1:200) for 1 h at room temperature. The slides were washed with PBS again three times for another 30 minutes prior to being counterstained by 4',6-diamidino-2-phenylindole (DAPI) for 2 minutes. After three more washes, the slides were covered by microscopic glass with Anti-fade Mounting Medium for further study. The whole process was conducted with careful prevention of light.

### Immunohistochemical staining

**Figure 2:**
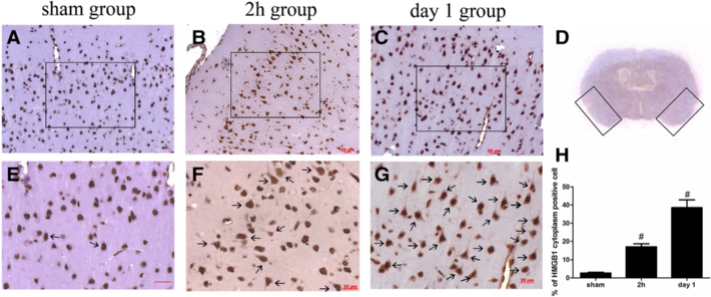
**Representative immunohistochemistry analysis of High-mobility group box 1 (HMGB1) in the brain cortex. (A)** HMGB1 expression in the sham group: HMGB1 could be observed in the nucleus in the sham group, cells were seldomly stained positive for cytosolic HMGB1. **(B, C)** HMGB1 staining in the 2-h and day-1 groups post subarachnoid hemorrhage (SAH), respectively. The quantity of cells positive for cytosolic HMGB1 obviously increased in the SAH groups at 2 h and day 1 after SAH. **(D)** Black boxes show the view of observation.** (E, F **and **G)** Enlarged images of **A**, **B** and **C**, respectively. Positive cells were defined as presenting buffy grains in cytoplasm as shown by arrows. Scalar bars present 20 μm. **(H)** Quantification of density of cells positive for cytosolic HMGB1. Values were obtained from averaging three sections per animal. SAH induced translocation of HMGB1 in the cortex near the subarachnoid space as early as 2 h post SAH. Bars represent the mean ± standard error (n = 6); ^#^*P* < 0.01 compared with the sham group.

Coronal blocks cut from 4 mm to 6 mm anterior to the groove between the forebrain and cerebellum in each rat brain were prepared for immunohistochemistry. The tissue was fixed with 4% paraformaldehyde and embedded in paraffin. The tissue sections (4 μm) were used for immunohistochemical staining (Figure 2D). The sections were deparaffinaged as usual and incubated with 3% H_2_O_2_ in PBS for 10 minutes. Sections were incubated with an anti-HMGB1 monclonal antibody diluted 1:500(Epitomics, Burlingame, CA, USA). Pilot experiments with blocking peptides were performed to validate the specificity of primary antibodies before the experiments. Negative controls were prepared by omitting the primary antibodies. Each of the sections was incubated with horseradish peroxidase (HRP)-conjugated goat anti-rabbit IgG diluted 1:500(Santa Cruz Bio-technology, Santa Cruz, CA, USA) for 60 minutes at room temperature. Diaminobenzidine (DAB) was used as the chromogen and counterstaining was done with hematoxylin. Three coronary sections in each coronal block sample with a minimum of 100 μm from the adjoining section were used for cell counting in each sample. The number of cytoplasmic HMGB1-positive cells was presented as the percentage of total cells in each visual field. Three randomly non-overlapping high-power areas (×400) per section were selected and observed in the cortex as shown in the black box in Figure 2D. Then mean percentage of cytoplasmic HMGB1-positive cells in the three views was regarded as the data for each section. The final average percentage of the three sections was regarded as the data for the sample. Six samples in each group, respectively, were used for statistical analysis. The percentage of HMGB1-positive cells was identified, calculated, and analyzed under the light microscopy by an investigator blinded to the grouping.

### Immunofluorescence staining

Immunofluorescence staining was performed according to our previous study in our laboratory [3]. Brain tissue was fixed with 4% paraformaldehyde overnight and dipped in 20% saccharose PBS for 2 days and then in 30% saccharose PBS for another 2 days to remove water in the tissue. Sections 6 μm in thickness were sliced and blocked with 5% normal FBS in PBS containing 0.1% Triton X-100 for 2 h at room temperature prior to incubation with anti-neuron-specific nuclear protein (NeuN) antibody (Millipore, MA, USA, 1:200) and anti-HMGB1 antibody diluted 1:500(Catalog number 2600-1,Epitomics, Burlingame, CA, USA) or anti-ionized calcium binding adaptor molecule 1 (Iba1) antibody (Abcam, MA, USA,1:200) and anti-HMGB1 antibody diluted 1:500(Epitomics) or anti-glial fibrillary acidic protein (GFAP) antibody (1:200, BD Science, Franklin Lakes, New Jersey, USA) and anti-HMGB1 antibody diluted 1:500(Epitomics) or anti-cleaved caspase-3 diluted 1:500(Cell Signaling Technology, Beverly, MA, USA) and NeuN antibody (Millipore, MA, USA, 1:200) overnight at 4°C. After sections were washed three times with PBS for 45 minutes, they were immunolabeled with proper secondary antibodies (Alexa Fluor 488 and Alexa Fluor 594, 1:200) for 1 h at room temperature. The slides were washed with PBS again three times for 45 minutes prior to being counterstained by DAPI for 2 minutes. After three further washes, the slides were covered by microscopic glass with Anti-fade Mounting Medium for further study. Pilot experiments with blocking peptides were performed to validate the specificity of primary antibodies before the experiments. Negative controls were prepared by omitting the primary antibodies. Fluorescence microscopy imaging was performed using ZEISS HB050 inverted microscope system and handled by Image-Pro Plus 6.0 software (Media Cybernetics, USA). and Adobe Photoshop CS5 (Adobe Systems, San Jose, USA).

### HMGB1 measurements of cell-conditioned medium

Cell-conditioned medium was ultrafiltered and analyzed by western blot. Briefly, cell-conditioned medium (4 ml) was ultrafiltered using a Centricon (10 kDa, 4 ml, Millipore, MA, USA) according to the Instrument Manual at 4,000 × g with a typical final concentrate volume of about 100 μl. Sometimes more ultrafiltration tubes were required because Hb in the medium sometimes blocked the hole in the Centricon. About one third of the final volume was subjected for western blot analysis as described above. The primary antibodies wasanti-HMGB1 diluted 1:500(Epitomics, Burlingame, CA, USA). Detection was performed using detection reagents (Catalog number NCI5079, Thermo Scientific, Rockford, IL, USA) and were exposed to an x-ray film kit (Thermo Scientific, Rockford, IL, USA).

### Statistical analysis

All data were presented as mean ± standard error of the mean (SEM). SPSS 17.0 was used for statistical analysis of the data. The measurements were subjected to one-way analysis of variance (ANOVA). Differences between experimental groups were determined by the Student *t*-test. A value of *P* < 0.05 was considered statistically significant.

## Result

### General observation

In all the experimental SAH animals (n = 72), 6 of 54 (11.1%) animals injected with blood died while no animals died in the sham group. All mortality occurred within 24 h of surgery. Two rats with SAH were excluded from the study because of too little blood in the prechiasmatic cistern but many blood clots in the frontal lobe instead. Compared to the sham group (Figure 1A), the blood clots could easily be found on surface of the temporal lobe and around the basilar arteries (Figure 1B to E). It was also demonstrated that the blood clot in the subarachnoid space disappeared gradually with time (Figure 1B to E). No blood clots were found in the saline control group or in rHMGB1 injected groups (Figure 1F to J), and no rats in the control group died, while 3 of 45 rats died within 24 h after injection of rHMGB1.

### HMGB1 expression in the sham-group brain

**Figure 3:**
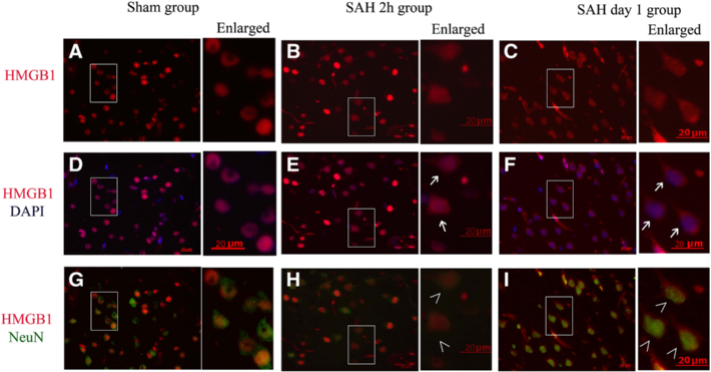
**Cytosolic expression of high-mobility group box 1 (HMGB1) in Neuron-specific nuclear protein(NeuN)-positive cells of cortex from animals with subarachnoid hemorrhage (SAH). (A, B, C) **HMGB1 immunostaining images obtained from the cortex of a sham **(A)**, a 2-h SAH **(B)**, and a day-1 **(C)** animal respectively. **(D, E, F)** Merged images of HMGB1 immunostaining (red) and 4',6-diamidino-2-phenylindole(DAPI) nuclear staining (blue). **(G, H, I)** Merged images of HMGB1 immunostaining (red) and NeuN immunostaining (green). Enlarged images on the right side of each panel highlight the increased number of NeuN-positive cells with cytosolic staining of HMGB1 in cortex from SAH animals. Arrows indicate the cytosolic HMGB1. The mark (>) indicates co-localization of cytosolic HMGB1 and NeuN, The results indicate that HMGB1 translocation occurred as early as 2 h and advanced in the process after SAH. Scale bars represent 20 μm.

**Figure 4:**
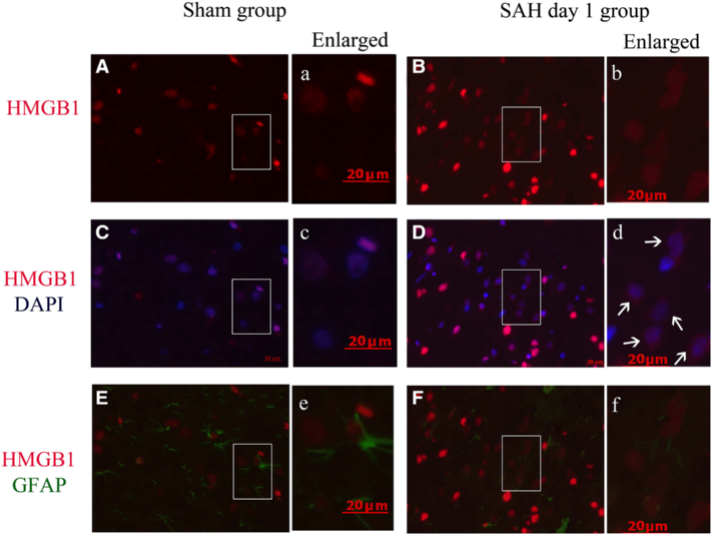
**Cytosolic expression of High-mobility group box 1 (HMGB1) in Glial fibrillary acidic protein (GFAP)-positive cells of cortex from animals with subarachnoid hemorrhage (SAH). (A, B)** HMGB1 immunostaining images obtained from cortex of a sham **(A)**, and a day-1 **(B)** animal respectively. **(C, D)** Merged images of HMGB1 immunostaining (red) and 4',6-diamidino-2-phenylindole (DAPI) nuclear staining (blue). **(E, F)** Merged images of HMGB1 immunostaining (red) and GFAP immunostaining (green). Enlarged images **(a, b, c, d, e, f)** on the right side of each panel also highlight the increased number of cytosolic HMGB1-positive cells in cortex from SAH animals while few cytosolic HMGB1-positive cells were also positive for GFAP. Arrows indicated the cytosolic HMGB1. The results indicated that astrocytes were not the main source of released HMGB1, at least at this early time point. Scale bars represent 20 μm.

**Figure 5:**
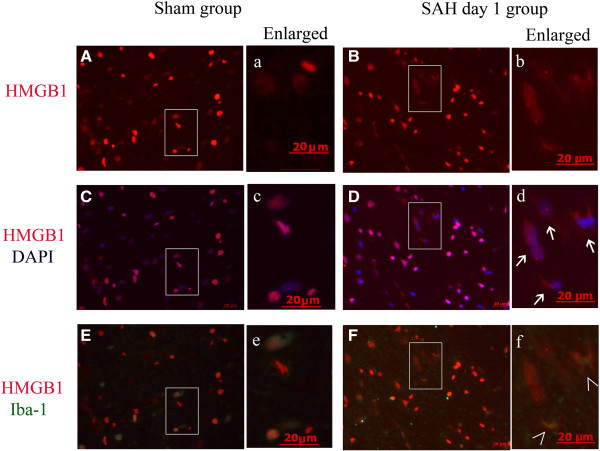
**Cytosolic expression of High-mobility group box 1 (HMGB1) in ionized calcium binding adaptor molecule 1 (Iba1)-positive cells of cortex from animals with subarachnoid hemorrhage (SAH). (A, B)** HMGB1 immunostaining images obtained from cortex of a sham **(A)** and a day-1 SAH **(B)** animal. **(C, D)** Merged images of HMGB1 immunostaining (red) and 4',6-diamidino-2-phenylindole (DAPI) nuclear staining (blue). **(E, F)** Merged images of HMGB1 immunostaining (red) and Iba1 immunostaining (green). Enlarged images **(a, b, c, d, e, f)** on the right side of each panel highlight the increased number of cytosolic HMGB1-positive cells (arrows) in cortex from SAH animals and some cytosolic HMGB1-positive cells were also positive for Iba1(shown as >). Scale bars represent 20 μm. Arrows indicat the cytosolic HMGB1. The mark (>) indicates co-localization of cytosolic HMGB1 and Iba-1. The results indicate that microglia were also a source of extracellular HMGB1. Scale bars represent 20 μm.

In the sham-group rat-brain coronal sections, HMGB1 was observed to be widely expressed in the nuclei of brain cells (Figure 2A), in either NeuN, GFAP, or Iba-1-positive cells (Figures 3D, 4C and 5C).

### Subarachnoid hemorrhage induction induces HMGB1 translocation and release in brain cells

**Figure 6:**
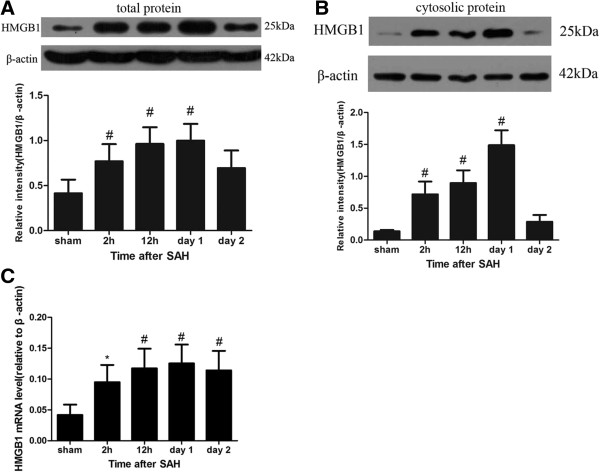
**Expression of High-mobility group box 1 (HMGB1) protein and mRNA level in brain cortex after subarachnoid hemorrhage (SAH). (A)** Western blot analysis of HMGB1 expression in the total protein extraction of the cortex after SAH showed that the HMGB1 protein level was significantly increased as early as 2 h and peaked approximately on day 1. **(B)** Western blot analysis of HMGB1 in the cytosolic protein fraction of the cortex. The quantity of HMGB1 protein in cytosolic protein fraction was also detected to increase in the 2-h, 12-h, day-1 and day-2 groups, which indicated that the HMGB1 translocation occurred as early as 2 h after SAH onset. **(C)** Real-time PCR analysis of HMGB1 mRNA level demonstrated the HMGB1 mRNA level was also up-regulated as early as 2 h after SAH. Bars represent the mean ± standard error (n = 6, each group). ^#^*P* < 0.01 compared with the sham group; *P < 0.05 compared with sham group.

HMGB1 was reported as a late responding signal molecule in sepsis [12]. Individual study indicated that HMGB1 level was increased in the late stage of SAH [13]. Little is known about the role of HMGB1 in the early stage of SAH. Thus, we examined a series of early time points in the rat SAH model to obtain a full view of HMGB1 protein level and location changes after SAH. Firstly, through western blot analysis of total tissue extracts, the level of HMGB1 protein increased significantly as early as 2 h after experimental SAH onset and peaked on day 1 post SAH when compared to the sham group(2 h, *P* < 0.05; 12 h, *P* < 0.01; day 1, *P* < 0.01; day 2, *P* < 0.05) (Figure 6A). To identify whether the increased level of HMGB1 protein was transferred from nucleus to cytoplasm, nuclear protein fraction and cytosolic protein fraction were extracted separately (see Method and material). HMGB1 protein level in the cytosolic protein fraction was detected to significantly increase as early as 2 h after SAH induction (2 h, *P* < 0.01; 12 h, *P* < 0.01; day 1, *P* < 0.01) (Figure 6B). The above results showed that SAH could cause significant increased production and translocation of HMGB1 protein in the brain cortex as early as 2 h post injury.

Through quantitative real-time PCR analysis, the mRNA level of HMGB1 in SAH groups was identified to increase compared to the sham group. In detail, low level mRNA of HMGB1 could be detected in the sham group while the HMGB1 mRNA expression was significantly higher in a time-dependent manner, similar to western blot in the SAH groups. A statistical difference was found not only between the sham and 2-h group (*P* < 0.05) but also between the sham group and the 12-h, day-1 groups (*P* < 0.01, respectively) (Figure 6C). The above results showed that SAH could cause significant active production of HMGB1 in the brain cortex as early as 2 h post injury.

HMGB1 translocation and release from brain cells was also confirmed by immohistochemistry study. The extra-nucleus or cytoplasm-positive HMGB1 staining cells were regarded as HMGB1-positive cells. As Figure 2B shows, HMGB1 translocation in the cortex occurred as early as 2 h after SAH. This result is in agreement with our western blot result (Figure 6B). The amount of cytoplasm HMGB1-positive cells could be observed to increase significantly in representative cortex from the 2-h (mean = 17.03%) to day-1 group (mean = 38.57%) (Figure 2F,G) compared to the sham group (mean = 2.63%). Semi-quantitative analysis showed that there was an obvious difference between the sham group and the 2-h, day-1 group (*P* < 0.01) (Figure 2H).

### Early HMGB1 translocation induced by SAH occurred mostly in neurons in brain tissue

Double immunofluorescent staining was performed for HMGB1 and NeuN, Iba1 or GFAP to identify the cell types in which HMGB1 translocation occurred after SAH. We examined several time points in the SAH rat model to confirm the translocation of HMGB1 and how early it occurred after SAH. As Figure 3 shows, most cytosolic HMGB1-positive cells were also positive for NeuN-staining. In comparison with the sham group, SAH also induced HMGB1 translocation in cells positive for Iba-1 (Figure 5). Few cells were found to stain positive for cytoplasmic HMGB1 and GFAP (Figure 4). These results suggest that HMGB1 translocation mainly occurred in neurons in the injured cortex following SAH. Meanwhile a small number of Iba-1-positive cells started to secrete HMGB1 in the early phase following SAH. These findings may in part reflect the selective vulnerability of neurons to the injury and suggest that neurons might be one of the main sources of the released HMGB1, at least in this early phase.

### Both passive and positive release of HMGB1 are involved in the increased level of HMGB1

**Figure 7:**
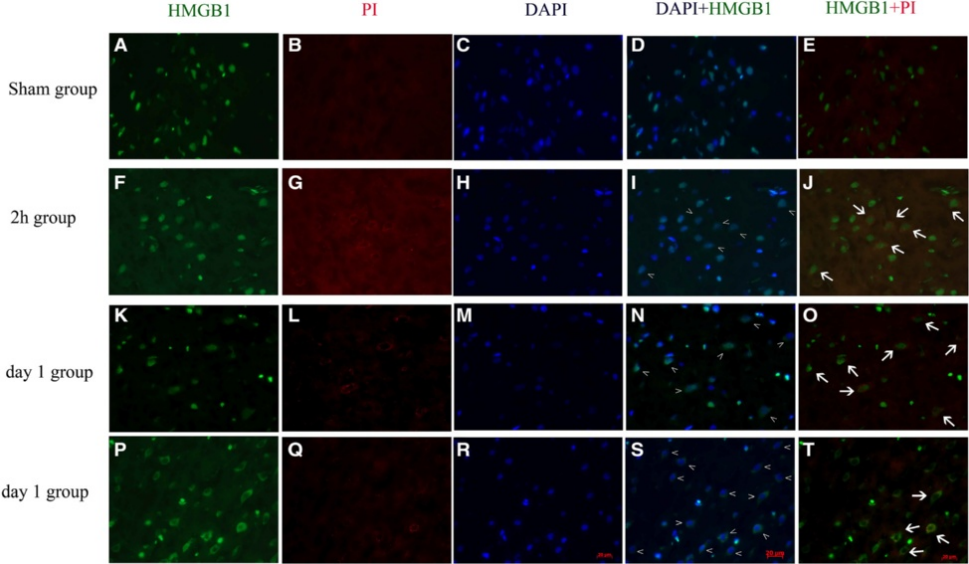
**Representative photomicrographs show brain cortex double-staining of immunofluorescent (High-mobility group box 1 (HMGB1), green) and PI staining (red) in the sham (A - E), 2-h (F - J), day -1 (K - O), and another day-1 (P - T) group.** The nucleus was counterstained with 4',6-diamidino-2-phenylindole (DAPI) (blue) in the same view in each section. **(D, I, N, S)** Merged images of HMGB1(green) and DAPI (blue); **(E, J, O, T)** merged images of propium iodide (PI) (red) and HMGB1 (green). Compared with the sham group **(D)**, the subarachnoid hemorrhage (SAH) groups **(I, N, S)** show translocation of HMGB1 from nuclear to cytoplasm (the mark (>) indicates the cytosolic HMGB1). Few PI-positive cells could be observed in the sham group **(B, E)** while patches of cells positive for PI could be detected in the SAH groups **(G, L)**. Overlapping images **(J, O)** showed that most cells were positive for both cytosolic HMGB1 and PI staining (arrows indicate the co-localization of cytosolic HMGB1 and PI). However, positive staining for cytosolic HMGB1 but not positive for PI staining could also easily found in the day-1 group post SAH **(T)**. Scale bar: 20 μm. These results support the theory that both passive and active release of HMGB1 are involved in the translocation process.

To give support to the hypothesis that both passive and positive release of HMGB1 were engaged in HMGB1 translocation. PI staining was employed to distinguish the dead cells and survival cells [21]. As Figure 7 shows, seldom cells were stained for PI in the sham group while PI-positive cells could be easily found in the 2-h and day-1 groups (Figure 7B,G,L,Q). Cells positive for PI and cytoplasmic HMGB1 were observed both in the 2-h and day-1 groups, which indicates that these cells were in an injured condition and HMGB1 might be released passively (Figure 7J,O) [9]. However, HMGB1 cytoplasm-positive cells that were not positive for PI were also detected in another representative view of the day-1 group (Figure 7T), which means these cytoplasmic HMGB1 were actively secreted. These results were also in agreement with our real-time PCR result.

### Extracellular HMGB1 induces inflammatory cytokines and cleaved caspase3 activity *in vivo*

**Figure 8:**
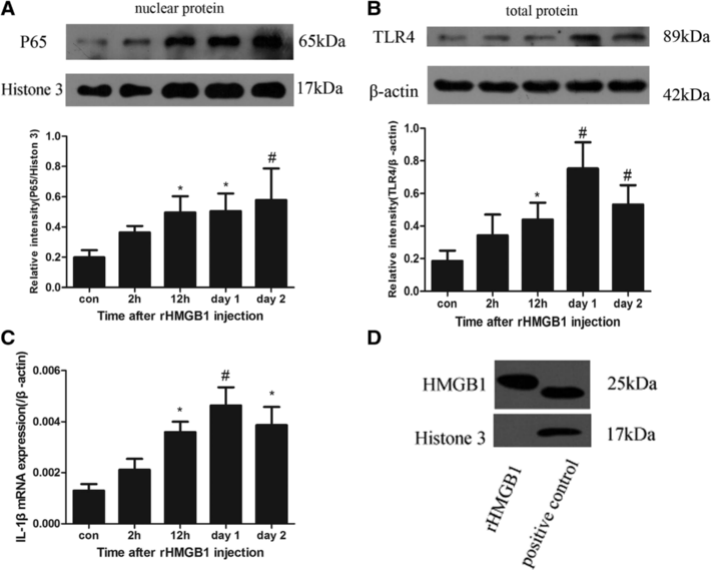
**Addition of recombinant High-mobility group box 1 (rHMGB1) in the subarachnoid space triggered inflammatory response *in vivo*.** NF-κB was measured by the western blot of its P65 subunit in the nucleus. Toll-like receptor (TLR)4 protein level was also detected by western blot analysis. IL-1β was measured by real-time PCR. **(A)** rHMGB1 up-regulated P65 subunit protein level in the nuclear protein in cortex cells; *P* < 0.05 between the 12-h, day-1 group and control group, *P* < 0.01 between the day-2 group and control group. **(B)** rHMGB1 increased TLR4 protein level. *P* < 0.05 between the 12-h and control group, *P* < 0.01 between the day-1, day-2 group and control group. **(C)** rHMGB1 upregulated IL-1β mRNA expression in cortex cells: *P* < 0.05 between the 12-h, day-2 group and control group, *P* < 0.01 between the day-1 group and control group. **(D)** Western blot analysis of histone 3 content in rHMGB1(left lane) and the nuclear protein extraction(right lane). The result could exclude histone 3 contamination in rHMGB1 products. Histone 3 was predominant composition of histone protein, thus, our result indicated the rHMGB1 used in the study had good purity. Bars represent the mean ± standard error (n = 6): *P < 0.05 compared with the control group; ^#^*P* < 0.01 compared with the control group.

**Figure 9:**
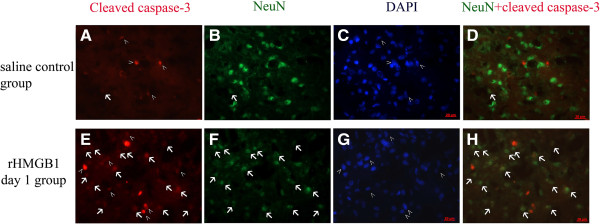
**Representative photomicrographs showed brain neurons double immunofluorescent staining for cleaved caspase 3 (red) and Neuron-specific nuclear protein (NeuN), a neuron cell marker (green) *in vivo *in the control (A to D) and recombinant High-mobility group box 1 (rHMGB1) treatment group (E to H).** The nucleus was counterstained with 4',6-diamidino-2-phenylindole (DAPI) (blue) in the same view in each section. **(D, H)** Merged images of cleaved caspase 3 (red) and NeuN(green). Compared with the control group **(A)**, more cleaved caspase 3-positive cells were detected in the cortex after rHMGB1 treatment **(E)**. Especially, overlapping images **(H)** showed that the number of cells positive both for cleaved caspase 3 and NeuN increased compared with control group **(D)**. The marks (>): profiles positive for cleaved caspase 3 and DAPI but negative for NeuN showed activation of caspase 3 in non-neuronal cells. Arrows: profiles positive for cleaved caspase 3 and NeuN showed activation of caspase 3 in neurons. Scale bar: 20 μm. These results indicate that rHMGB1 addition increased the cleaved-caspase 3 positive cells, especially the neurons. RHMGB1 might promote the cell apoptosis.

TLR4 is the important receptor of HMGB1. Western blot of total protein extraction demonstrated that rHMGB1 induced increased level of TLR4 (*P* < 0.05 between the control group and 12-h group; *P* < 0.01 between the control group and day-1, day-2 groups respectively) (Figure 8B). This result indicates that extracellular HMGB1 could initiate TLR4 signal pathway. NF-κB activity is usually measured by electrophoretic mobility shift assay (EMSA) [22] and nuclear translocation of its main proinflammatory subunit P65 [23,24]. Thus, the protein level of P65 in the nucleus extract is usually used to evaluate the activity of NF-κB in our laboratory [25,26]. The nuclear extract was prepared for western blot analysis of NF-κB(P65). As Figure 8A shows, the P65 subunit in the nucleus was significantly increased in the 12-h, day-1, day-2 groups after injection of rHMGB1 (12 h, *P* < 0.05; day 1, *P* < 0.05; day 2, *P* < 0.01). The downstream inflammatory factor IL-1β, was also detected to be up-regulated by real-time PCR (12 h, *P* < 0.05; day 1, *P* < 0.01; day 2, *P* < 0.05) (Figure 8C). Cleaved caspase 3 staining, a possible marker of apoptosis, was used to evaluate the brain injury after rHMGB1 injection. Compared with the control group, the number of cells positive for cleaved caspase 3 and NeuN was increased, which suggested that rHMGB1 might be a harmful molecule for brain cells, especially for neurons (Figure 9) and extracellular HMGB1 might contribute to the brain injury after SAH.

### Massive HMGB1 release from Hb-incubated neurons

**Figure 10:**
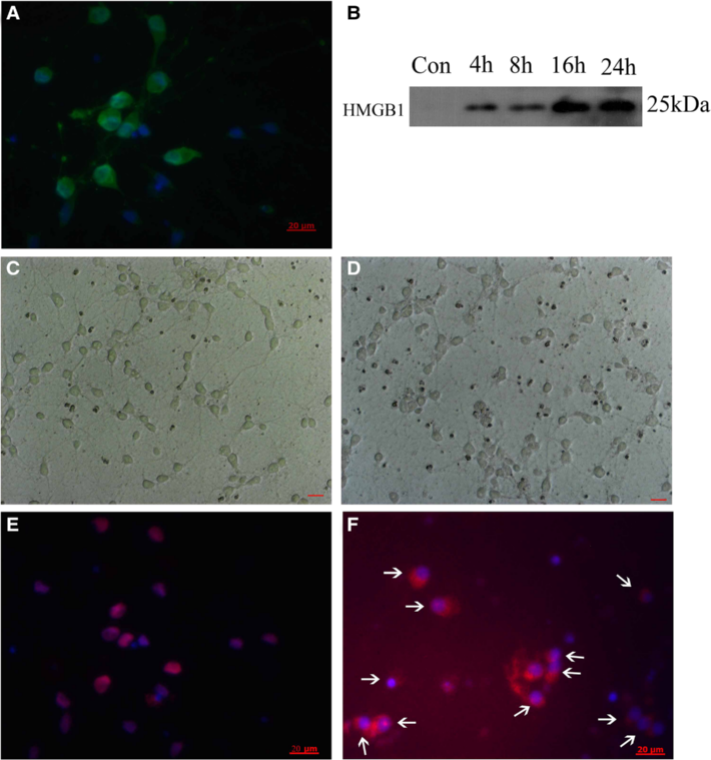
**Representative photomicrographs showed brain neurons double immunofluorescent staining for High-mobility group box 1 (HMGB1) (red) and Neuron-specific nuclear protein (NeuN), a neuronal cell marker (green) *in vitro *in the control (C, E) and Hb incubation group (D, F). (A)** Immunofluorescence staining shows more than 98% cells were positive for both Neuron and 4',6-diamidino-2-phenylindole (DAPI), which suggests the high percentage of Neuron cells in the primary cultured cells. **(B)** Result of western blot analysis of concentrated conditioned culture media, which showed that HMGB1 could be detected in the media. **(C, D)** Images of cultured neurons in light microscopy to watch its sharp; light micrograph of neurons shows cellular morphology in sham group **(C)** and Hb incubation group **(D)**. **(E, F)** Merged images of HMGB1(red) and DAPI (blue) in cultured neurons. Compared with the sham group **(E)**, the Hb incubation groups **(F)** showed translocation of HMGB1 in cytoplasm (as shown by white arrows). Scale bar: 20 μm.

To confirm the neuronal susceptibility of early HMGB1 translocation, primary cortical neuron culture was subjected to Hb treatment and HMGB1 translocation was detected by immunofluorescence staining and western blot. Immunofluoresecnce staining showed more than 98% cells were positive for both NeuN (green) and DAPI (blue) which suggested the high percentage of neurons in the primary cultured cells (Figure 10A). Through western analysis, HMGB1 was undetectable in medium from untreated cells. However, HMGB1 was found to accumulate in culture medium of Hb-incubated neurons (Figure 10B). A phase-contrast micrograph of neurons demonstrated cellular morphology of untreated (Figure 10C) and 24-h Hb (Figure 10D). Furthermore, HMGB1 was detected as nucleus-positive in The control group (Figure 10E) and cytoplasm-positive in Hb-treated groups (Figure 10F). This result indicates that HMGB1 in neurons was in a process from nucleus to extracellular after Hb incubation.

### Conditioned medium from Hb-treated neurons induced IL-1β in cultured mixed glial cells, which could be inhibited by HMGB1-specific inhibitor

**Figure 11:**
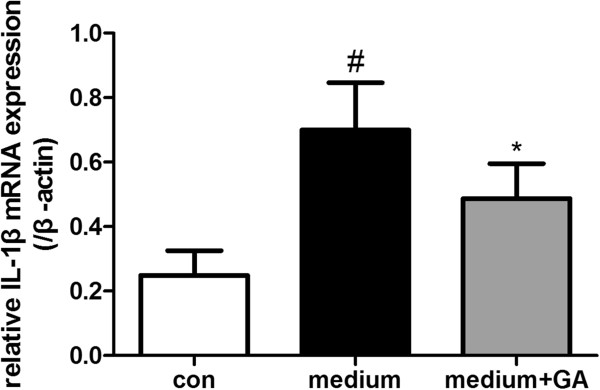
**Conditioned medium from Hb-treated neurons induced IL-1β in cultured mixed glial cells, which could be inhibited by High-mobility group box 1 (HMGB1)-specific inhibitor.** Cultured mixed glial cells were arranged into three groups. In the control group mixed glial cells were treated with control medium; in the medium group mixed glial cells were treated with neuron medium; in the medium + glycyrrhizic acid (GA) group, after mixed glial cells were treated with neuron medium, a special inhibitor of HMGB1 (GA) was added in the medium to silence the activity of HMGB1. IL-1β was measured by real-time PCR. Bars represent the mean ± standard error (n = 6), *P < 0.05 compared with the medium group; ^#^*P* < 0.01 compared with the control group.

To determine whether HMGB1 released from injured cultured neurons was biologically active as a pro-inflammatory mediator, we treated primary mixed glial cell cultures with conditioned medium from Hb-treated neurons. To remove residual Hb in supernatant, neurons were cultured in fresh DMEM after being exposed to Hb (20 μmol) for 2 h. As shown in Figure 11, conditioned medium robustly induced IL-1β mRNA expression in glial cells. However, IL-1β could be inhibited after treatment with HMGB1-specific inhibitor GA (*P* < 0.01 between the control and medium group; *P* < 0.05 between the medium group and medium + GA group). This result indicates that HMGB1 in the medium played an important role in activation of glial cells.

## Discussion

In this study, we demonstrated that (1) HMGB1 was translocated from the nucleus to the cytoplasm and released from neurons as early as 2 h after SAH associated with a significant upregulation of protein and mRNA level; (2) both passive and active release of HMGB1 were involved in the process of HMGB1 translocation; (3) rHMGB1 or HMGB1 released from neurons could induce inflammatory response, and (4) extracellular HMGB1 contributed to the early brain injury after SAH.

Previous studies have demonstrated that HMGB1 could be released from necrotic cells passively or secreted actively from immune cells or non-immune parenchymal cells, such as hepatocytes,in ischemia [27,28]. In our study, we demonstrated that HMGB1 was released from cortex near the blood clot as early as 2 h after SAH onset according to our western blot and immunhistochemistry results. Friedrich *et al*. showed that cortex cell death occurred as early as 10 minutes after SAH [14]. As a consequence, passive release of HMGB1 was possibly initiated by damaged cellular integrity. This hypothesis was also supported in our study, which showed co-location of PI staining and cytosolic HMGB1 staining (Figure 7J). However, active secretion of HMGB1 was also supported by up-regulated mRNA and protein levels of HMGB1 (Figure 6A to C) in our study. Further, positive staining for cytoplasmic HMGB1 but negative for PI staining also support this theory (Figure 7S,T). Our research was consistent with research into liver ischemia, which has shown that the liver parenchymal cell, liver cells could actively release HMGB1 under ischemic conditions [27,28]. Thus, it is possible that both passive and active releases of HMGB1 are involved in the process of HMGB1 translocation. However, co-immunoprecipitation might be the best method to make sure whether cytoplasmic HMGB1 was passive or actively released [28]. Previous study indicated that both passive and active release of HMGB1 have cytokine activity and could trigger inflammatory response [29], thus co-immunoprecipitation of HMGB1 was not performed in this study.

Extracellular HMGB1 was regarded as a member of the DAMP family [30]. HMGB1 functions as a pro-inflammatory factor through its receptors, including TLR2, TLR4 and RAGE [11]. Conceivably, up-regulated expression of HMGB1 receptors after SAH might enhance the sensitivity of brain cells to HMGB1. Both TLR4 and RAGE were reported to ascend early after SAH in recent studies [4,26,31]. Although interaction of HMGB1 with these receptors signals through different pathways, they ultimately promote the NF-κB/P65 translocation to nucleus and activation of NF-κB, which activates the transcription of downstream pro-inflammatory genes (IL-1β, TNF-α) [11]. Addition of rHMGB1 in the subarachnoid space up-regulated TLR4, P65 protein levels and downstream inflammatory response in this study, which confirmed that extracellular HMGB1 could trigger the inflammatory response and the TLR4/NF-κB signal pathway might be one of the activated inflammatory pathway in HMGB1-induced inflammatory response.

Previous study has shown that the most important receptors of HMGB1, TLR4 and RAGE are up-regulated 4 h and 6 h after SAH, respectively. IL-1β reaches its peak on day 1 after SAH [3,31]. In this study HMGB1 translocation was detected as early as 2 h post injury. Furthermore, *in vivo*, we observed a two-fold induction of IL-1β mRNA level after subarachnoid injection of rHMGB1 (Figure 8C). Previous studies also demonstrated that HMGB1 incubation in glia or neuron cells *in vitro* induce 2- to 4-fold inflammatory factor mRNA induction [10,22]. The data reported here showed the translocation of HMGB1 preceded the increase of other cytokines, which indicates that HMGB1 may act as an early upstream initiator of inflammation after SAH.

Immunohistochemical and immunofluorescence staining results showed most cells positive for cytoplasmic HMGB1 were also positive for NeuN. A small number of cytoplasmic HMGB1-positive cells were found to be microglia. Neurons seem to be susceptible after brain injury and the main source of extracellular HMGB1. Actually, not only in the SAH model *in vivo* and *in vitro*, but also in the ischemic brain, neurons seem to be the main source of HMGB1 in the early stage of brain injury [10]. Early-released HMGB1 from neurons might be the important early upstream factor in the following inflammatory response after SAH. To better understand whether neuron-derived HMGB1 could signal to mixed glial cells, medium from neurons, which had been stimulated by Hb, was used to culture the mixed glial cells (see detailed information in Method and material). The result demonstrates that HMGB1 released from neurons could initiate the inflammatory response in mixed glial cells. Thus, it could be supposed that HMGB1 released from neurons might influence neighboring glial cells and up-regulate the inflammatory factors, which could in turn stimulate brain cells to release more HMGB1 with an up-regulated mRNA level of HMGB1 [9]. This result suggests further that HMGB1 might be an early upstream factor in inflammation after SAH. Myeloid differentiation primary response protein 88 (downstream of TLRs) and RAGE were also reported to be up-regulated mainly in neurons after SAH [3,26]. Our previous study *in vitro* showed addition of rHMGB1 could also increase MyD88 expression in protein and mRNA levels in cultured neurons with up-regulated inflammatory factors [22]. According to these studies, it seems that relative healthy neurons nearby could also be reactive cells for extracellular HMGB1 as well as glial cells. A small number of microglia was observed to release HMGB1 in the beginning. However, it was believed that more and more microglia would start to secrete HMGB1 as time developed, which was observed in the late stage in a previous study [13]. Interestingly, this phenomenon also exists in the late stage of the ischemic brain [32]. HMGB1 released from microglia might be responsible for the inflammation in the late stage.

Addition of rHMGB1 up-regulated cleaved caspase 3, a possible marker of apoptosis in the cortex, especially in neurons (Figure 9). Extracellular rHMGB1 seemed harmful for survival of brain cells. The explicit mechanism is not yet clear. The inflammatory response induced by rHMGB1 might be the possible reason. It is widely believed that inflammation in the early stage contributes to the brain injury and it has been confirmed that inhibition of NF-κB and downstream inflammatory factors confer protection in the early stage [2,8,24]. Increased inflammatory factors, such as IL-1β and TNF-α, could mediate cell apoptosis and cell damage [33,34]. Thus, it was suspected that the rHMGB1 might accelerate brain injury through up-regulated inflammation. How the rHMGB1 in the subarachnoid space influenced the cortex parenchymal cells is not yet clear. Maybe it shared a similar process to the intraventricular injection of lipopolysaccharide [34] or the small molecule HMGB1 might be able to easily infiltrate the pia mater because of its small molecular weight.

Rodex modification of 106 cysteine in the HMGB1 molecule might be critical for its cytokine activity. Oxidization of the cysteine at position 106 in HMGB1 could suppress the pro-inflammtory activity [35,36]. However, although together with ROS, HMGB1 retains its activity during the inflammatory process in a glutamate-treated-neurons model, oxygen-glucose deprivation model [10] and an N-methyl-D-aspartic acid (NMDA)-treated model [32]. Further research using tandem mass spectrometric analysis indicated that the predominant form of HMGB1 during the inflammatory process is not oxidized. When the inflammation was resolved, the form of HMGB1 was oxidized [35]. As shown in Figure 11, although ROS could be provided by hemoglobin, HMGB1 cytokine activity still existed and triggered inflammation in glial cells. Furthermore, ROS also damages cells itself and upregulates HMGB1 release [37]. Thus oxidization could not silence all the HMGB1 because the treatment target of HMGB1 could alleviate the inflammatory response [38]. Early use of HMGB1 inhibitor, such as GA, might be a good choice for stopping the harmful inflammatory response.

Combining the research listed above, we could speculate that early-released HMGB1 from neurons after SAH onset might trigger inflammation in neurons [22], and glial cells nearby with their MyD88-level up-regulated [3]. Increased levels of inflammatory factors might trigger more cells to actively secrete HMGB1 [9]. Up-regulated inflammation contributes to the BBB breakdown, brain edema, cell apoptosis and death. Extracellular HMGB1 might be the early key mediator that mediates cross-talk between injured cells and relative healthy cells around damaged tissues [16].

The effects in clinic trials targeting a single signal in the inflammatory pathway are not as good as expected [39,40]. Inflammation is such a complex pathophysiological process, that treatment simply targeting a single molecule or receptor is difficult to determine. It is important to find out the source of the inflammatory response. Our research points out that HMGB1 might be the early key mediator in the process of SAH and a novel potential target for treatment [41].

### Summary

As mentioned above, HMGB1 is massively released from neurons early after SAH onset. Passive and active releases are involved in the translocation process. Extracellular HMGB1 represented a pro-inflammatory role and contributed to brain injury. HMGB1 might be the major upstream inflammatory mediator, which might be a potential treatment target.

## Abbreviations

ANOVA: analysis of variance; BBB: blood brain barrier; CNS: central nervous system; CSF: cerebrospinal fluid; DAB: diaminobenzidine; DAMP: damage-associate molecular pattern; DAPI: 4',6-diamidino-2-phenylindole; DMEM: Dulbecco's modified Eagle's medium; FBS: fetal bovine serum; EMSA: electrophoretic mobility shift assay; GFAP: Glial fibrillary acidic protein; Hb: hemoglobin; HMGB1: High-mobility group box 1; Iba1: Ionized calcium binding adaptor molecule 1; IL-1β: interleukin 1β; MyD88: Myeloid differentiation primary response gene; NeuN: Neuron-specific nuclear protein; NF-κB: Nuclear factor kappa B; NMDA: N-methyl-D-aspartic acid; PBS: phosphate-buffered saline; PCR: polymerase chain reaction; PI: propidium iodide; RAGE: receptors for advanced glycation end-products; rHMGB1: recombinant High-mobility group box 1; ROS: reactive oxygen species; SAH: subarachnoid hemorrhage; SD: Sprague-Dawley; TLR: toll-like receptor; TNF-α: tumor necrosis factor-alpha.

## Competing interests

All the authors declared that they have no competing interests.

## Authors’ contributions

QS and Y-CH designed the studies, carried out the surgery, physiology studies, immunohistochemical studies, and data analysis, and wrote the manuscript. WW designed the additional experiment and contributed much to the revised manuscript. HL and SL conducted the neuronal cell culture. DZ, W-DL and J-HZ contributed to the Western blotting and immunohistochemical analysis. BM and LZ contributed to the real-time PCR analysis. WL and M-LZ contributed to the design and analysis of the study. C-HH contributed to the design and analysis of the study and wrote the manuscript. All authors read and approved the final manuscript.

## Authors’ information

Qing Sun, Wei Wu and Yang-Chun Hu co-first authors.
